# Non-toxigenic environmental *Vibrio cholerae* O1 strain from Haiti provides evidence of pre-pandemic cholera in Hispaniola

**DOI:** 10.1038/srep36115

**Published:** 2016-10-27

**Authors:** Taj Azarian, Afsar Ali, Judith A. Johnson, Mohammad Jubair, Eleonora Cella, Massimo Ciccozzi, David J. Nolan, William Farmerie, Mohammad H. Rashid, Shrestha Sinha-Ray, Meer T. Alam, J. Glenn Morris, Marco Salemi

**Affiliations:** 1 Emerging Pathogens Institute, University of Florida, Gainesville, USA; 2 Department of Environmental and Global Health, College of Public Health and Health Profession, University of Florida, Gainesville, Florida, USA; 3 Department of Pathology, Immunology and Laboratory Medicine, University of Florida, Gainesville, USA; 4 Department of Infectious, Parasitic and Immunomediated Diseases, Istituto Superiore di Sanità, Rome, Italy; 5 Department of Public Health and Infectious Diseases, Sapienza University of Rome, Rome, Italy; 6 University Hospital Campus Bio-Medico, Italy; 7 Interdisciplinary Center for Biotechnology Research, University of Florida, Gainesville, Florida, USA; 8 Department of Medicine, College of Medicine, University of Florida, Gainesville, Florida, USA

## Abstract

*Vibrio cholerae* is ubiquitous in aquatic environments, with environmental toxigenic *V. cholerae* O1 strains serving as a source for recurrent cholera epidemics and pandemic disease. However, a number of questions remain about long-term survival and evolution of *V. cholerae* strains within these aquatic environmental reservoirs. Through monitoring of the Haitian aquatic environment following the 2010 cholera epidemic, we isolated two novel non-toxigenic (*ctxA/B*-negative) *Vibrio cholerae* O1. These two isolates underwent whole-genome sequencing and were investigated through comparative genomics and Bayesian coalescent analysis. These isolates cluster in the evolutionary tree with strains responsible for clinical cholera, possessing genomic components of 6^th^ and 7^th^ pandemic lineages, and diverge from “modern” cholera strains around 1548 C.E. [95% HPD: 1532–1555]. Vibrio Pathogenicity Island (VPI)-1 was present; however, SXT/R391-family ICE and VPI-2 were absent. Rugose phenotype conversion and vibriophage resistance evidenced adaption for persistence in aquatic environments. The identification of *V. cholerae* O1 strains in the Haitian environment, which predate the first reported cholera pandemic in 1817, broadens our understanding of the history of pandemics. It also raises the possibility that these and similar environmental strains could acquire virulence genes from the 2010 Haitian epidemic clone, including the cholera toxin producing CTXϕ.

The exogenous introduction of toxigenic *Vibrio cholerae* O1 to Hispaniola putatively via United Nations peacekeepers in 2010 garnered global attention^1^. Despite the identification of the source of this introduction, the history of cholera in Hispaniola remains elusive. Epidemic cholera was first reported throughout the Caribbean in the 19^th^ century, and while outbreaks in Haiti were not documented, the existence of the pathogen in this setting cannot be totally ruled out^2^. Surveillance of the aquatic environment in Haiti following the 2010 epidemic has identified toxigenic *V. cholerae* O1 that are clearly derived from the circulating epidemic strain, as well as potentially indigenous non-toxigenic O1 and non-O1/non-O139 strains^3^,^4^,^5^,^6^. These latter findings have led to conjecture regarding autochthonous *V. cholerae* in Haitian aquatic environments, the sources of origin for these strains, and the possibility of novel toxigenic O1 and non-O1/O139 strains arising through lateral gene transfer of the CTXϕ^7^,^8^. The identification of non-toxigenic *V. cholerae* O1, therefore, raises fundamental questions about the complex history of cholera in the region.

Aquatic environmental reservoirs play a critical role in the transmission of *V. cholerae* to humans, and the reservoirs can also significantly impact evolutionary dynamics^9^. Environmental *V. cholerae* isolates are typically antigenically diverse and do not commonly possess cholera toxin genes or toxin coregulated pilus (TcpA)^10^. However, environmental strains are epidemiologically and evolutionarily important as they may serve as a donor of genetic material through natural transformation. Similarly, they may acquire the O1 antigen and virulence genes through horizontal gene transfer^11^. Historically, the O1 biotype has been primarily associated with human disease, with both the 6^th^ and 7^th^ cholera pandemics likely arising through horizontal gene transfer among descendants of a common *V. cholerae* O1 El Tor ancestor^12^. Non-toxigenic *V. cholerae* O1 strains have previously been reported in the Caribbean, South America, and Australia associated with clinical cases and the environment^12^. These strains tend to phylogenetically cluster more closely with other O1 strains of the El Tor and classical lineage. They may also possess diverse genomes in terms of genomic islands, antibiotic resistance, and virulence genes^13^,^14^. Through ongoing monitoring of Haitian aquatic environments during the current epidemic, we identified two environmental non-toxigenic (*ctxA/B* negative) *V. cholerae* O1 strains possessing classical *tcpA* genes and genomically similar to the O395 classical strain^3^. To further investigate these relationships, the genomes were closed using PacBio long-read sequencing data and phylogenetically compared to representative clinical and environmental O1 and non-O1/O139 *V. cholerae* strains, including the recently published 2^nd^ pandemic classical strain. Bayesian molecular clock analysis estimated the divergence date around 1548 C.E., consistent with the timeframe of the first European settlements in Hispaniola.

## Results and Discussion

### Isolation and characterization of *V. cholerae* strains

In 2012, we isolated two *V. cholerae* O1 strains, 2012Env-9 and 2012Env-390, from Haitian aquatic environments. Sampling sites were in estuaries of two separate rivers emptying into Port-au-Prince Bay separated by a linear distance of 5.4 miles. 2012Env-9 was isolated from the mouth of the Momance River (18°33′49.8″N 72°34′15.2″W) in La Salle in April, 2012, and 2012Env-390 was isolated from the mouth of a small river in Gressier (18°32′47.6″N 72°31′35.4″W) in September of the same year. The temporal and spatial variation in collection is consistent with wider diffusion of this strain in Haitian waterways. However, despite ongoing aquatic environment monitoring for toxigenic *V. cholerae* by at least four groups^4^,^6^,^15^,^16^, it is difficult to assess the true prevalence of this non-toxigenic O1 lineage in Haitian aquatic environment since the focus has largely been on identifying toxigenic strains. Genetic analysis showed that both were cholera-toxin (*ctx*) negative O1 strains and were positive for *ompW*, *toxR*, and *tcpA*^CL6^. While non-O1/O139 isolates harboring the *tcpA* gene have frequently been recovered in Haitian aquatic environmtents^4^, biotypic analysis demonstrated these non-O1/O139 isolates did not match either the typical classical or El Tor biotype. Instead, they possessed traits consistent with a hybrid of classical and El Tor biotypes (Supplemental Table 3).

### PacBio Sequencing and Genomic Comparison

**Figure 1:**
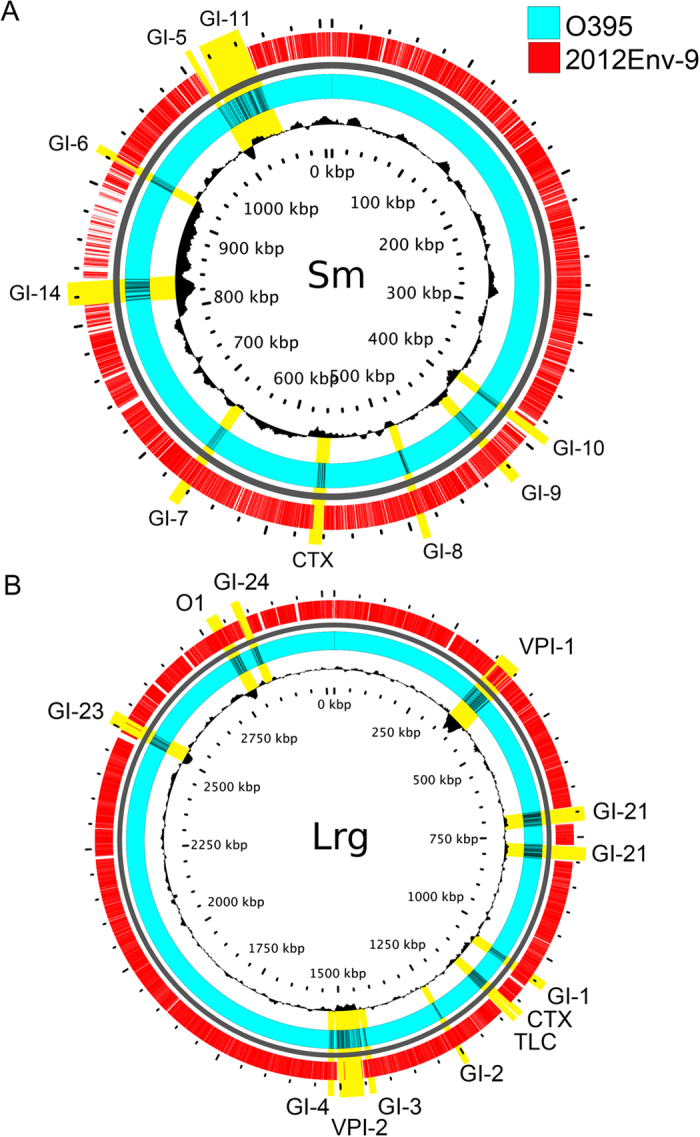
BLAST atlas of 2012Env-9 compared to *V. cholerae* O395 classical strain (NC_009456 and NC_009457). The black ring in the center demonstrates the GC content of the O395 genome. Blue and red rings represent the O395 and 2012Env-9 genomes, respectively, measured in kilobases. Notable genomic regions including cholera toxin phage (CTX), genomic islands (GI), vibrio pathogenicity islands (VPIs), and toxin-linked cryptic (TLC) are labeled and highlighted in yellow. (**A**) Small chromosome (**B**) Large chromosome.

Assembly and annotation of PacBio long read data fully resolved both the large large (Chr-1) and small (Chr-2) chromosomes of 2012Env-9 and 2012Env-390. For 2012Env-9 the size of the chromosomes was 2,937,102 bp with 2,552 coding sequences and 1,124,711 bp with 1,052 coding sequences for the Chr-1 and Chr-2, respectively. For 2012Env-390 the sizes of the chromosomes were 2,925,021 with 2,542 coding sequences and 1,125,906 bp with 1,055 coding sequences for the Chr-1 and Chr-2, respectively. Annotated sequences for 2012Env-9 (CP012997, CP012998) and 2012Env-390 (CP013013, CP013014) have been deposited in GenBank. BLAST comparison of genomic regions of interest between 2012Env-9 and classical strain O395 identified the presence of genomic islands (GIs) 1–4, related to membrane proteins, motility, and oxidative stress response, as well as the O antigen region and VPI-1 on chromosome 2 (Fig. 1). While GIs 7–9 were found to be present on Chromosome 1, VIP-2 was confirmed to be absent. The SXT/R391-family antibiotic resistance element, a ~100-kb integrative conjugative element (ICE), predicted to have been acquired by pandemic cholera between 1978–1984, was found to be absent in both strains^13^,^17^.

In the 2012Env-9 and 2012Env-390 genomes, CTXϕ was not present at the El Tor site, between toxin linked cryptic (TLC) and RTX on chromosome 1, or at either of the classical sites, between genes VCO395_0503 (VCA0569) and VCO395_0513 (VCA0570). The deletion in the 2012Env-9/390 genomes encompasses CTXϕ and satellite phages TLCϕ and RS1ϕ, which are present in canonical toxigenic O1 strains^18^. The absence of CTXϕ core genes (*cep*, *orfU*, *ace*, and *zot*) and RS1 element (*rstR*, *rstA*, *rstB*, and *rstC*) were confirmed through BLAST searches of 2012Env-9/390. Instead, at the El Tor sites of 2012Env-9/390, there was a 382 bp noncoding region immediately after *rtxA,* consisting of defective *dif* site and putative XerC and XerD binding sites (SFig. 1). The XerCD recombinase system is required for the sequential acquisition of CTXϕ and its satellite phages at the *dif* site as previously described^19^. At the classical site, a 245 bp insertion, with high nucleotide identity to a transposase found in the RS2/CTX region of toxigenic strains, was present (SFig. 2).

**Figure 2:**
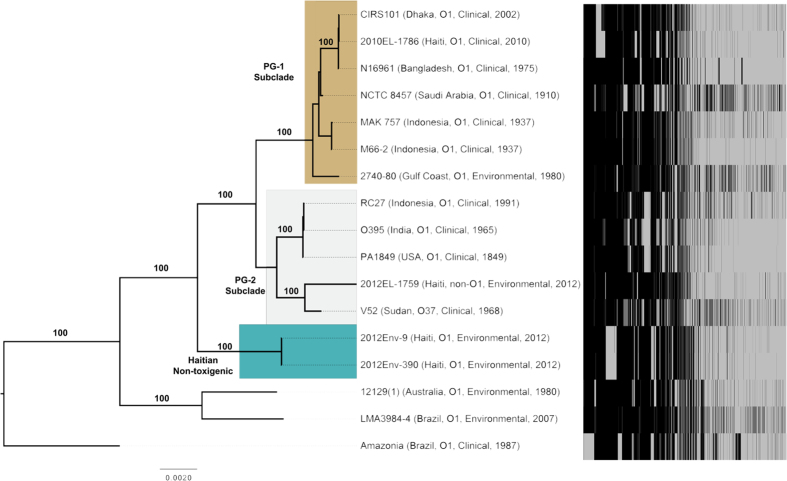
Evolutionary history of toxigenic and non-toxigenic *Vibrio cholerae* and accessory genomes. Maximum likelihood phylogeny of 17 environmental and clinical *V. cholerae* isolates inferred using RAxML analysis of 1838 genes (1,758,480 bp) in the core genome. Phylogeny was rooted using Path-O-Gen. Branches are labeled with bootstrap values and phylocore genome (PG) 1 and PG-2 are highlighted. Haitian non-toxigenic O1 strains are highlighted in green and are basal to the PG-2 subclade. To the right of the taxa names are the accessory genomes of the 17 toxigenic and non-toxigenic *V. cholerae* strains represented by a heatmap. Black represents the presence of a gene. Singletons (i.e., those genes only present in one isolate) were excluded from the heatmap.

Accessory genomes were then compared among 17 toxigenic and non-toxigenic *V. cholerae* O1 strains, specifically focusing on members of the phylocore genome (PG) subclades PG-1 and PG-2^12^ as well as other clinical and environmental non-toxigenic O1 strains. Clinical isolates were selected that represent the major waves of the 7^th^ pandemic and available strains from previous pandemics. Additionally, while diverse populations of non-O1 *V. cholerae* strains exist in aquatic environments of both cholera endemic and non-endemic regions, O1 strains are less frequently found in aquatic environments outside of cholera endemic regions. Therefore, we selected environmental O1 isolates for comparison that were genetically distinct from the contemporaneous epidemic clone. This comparison identified 6,953 genes with 1,933 genes shared among all strains (i.e., core genome) and 2,182 singleton genes possessed by only one strain (Fig. 2).

### Phylogenetic Analysis

The relationship among the core genomes of O1 and non-O1/O139 *V. cholerae* strains was investigated through phylogenetic analysis. We also individually assessed genealogies of three distinct genomic regions that putatively arose through lateral gene transfer: the oligosaccharide (OS) core region and O antigen region, comprising the major components of the lipopolysaccharide (LPS) as well as the VPI-1. First, the absence of substitution saturation, which decreases phylogenetic signal, was confirmed using several methods. Single nucleotide polymorphism (SNP) densities were well distributed across the core genome (SFig. 3) and transitions and transversions among 1+2 and 3 codon positions increased linearly with the genetic distance, with transitions being higher than transversions (SFig. 4). The Xia test also demonstrated an absence of substitution saturation (index of substitution saturation (*I*_*ss*_) 0.07 ≪ *I*_*ss*_ critical (*I*_*ss.c*_) 0.84). Likelihood mapping analysis of the core genome, OS region, VPI-1, and O antigen region all demonstrated sufficient phylogenetic signal with <20% of quartet topologies falling into the center of the likelihood map (i.e., unresolved or “star-like” topologies) (SFig. 5).

**Figure 3:**
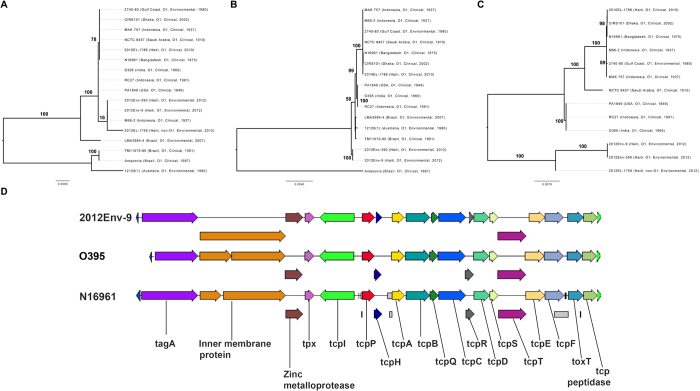
Genomic and phylogenetic comparison of *V. cholerae* O1 isolates. (**A**) Maximum likelihood (ML) phylogeny of the oligosaccharide (OS) region inferred from a four gene, 4,383 bp alignment (VC0227, VC0234, VC0236, VC0239). Bootstrap values are labeled on branches and tips are labeled with date and location of collection. (**B**) ML phylogeny of O antigen region inferred from a 16 gene, 24,517 bp alignment (VC0241-VC0254, VC0259-VC0263). (**C**) ML phylogeny inferred from a 23 gene, 19,503 alignment of Vibrio Pathogenicity Island 1 (VPI-1) (VC0823-VC0834,VC0836-VC0845). (**D**) Organization of VPI-1 gene cluster compared among toxigenic 6^th^ and 7^th^ Pandemic strains O395 (Classical) and N16961 (El Tor) and non-toxigenic Haitian environmental strain 2012Env-9.

Phylogenetic analysis of 17 environmental and clinical *V. cholerae* isolates illustrated that Haitian environmental isolates 2012Env-9 and 2012Env-390 were basal to isolates in PG-1 and PG-2 clades, yet more closely related to Classical and El Tor biotypes than other O1 isolates from Brazil and Australia (Fig. 2). Interestingly, 2012EL-1759, a non-O1/O139 environmental strain from Haiti, is located on the PG-2 sub-clade, most closely related to O37 isolate V-52, which was collected in 1968 from Sudan. Accessory genomes of 2012Env-9 and 2012Env-390 varied distinctly from environmental O1 strains 12129 (1) and LMA3984-4 (Fig. 2). When core OS regions were compared among strains, Haitian O1 strains were found to be Type 1, clustering with other members of the PG clade (Fig. 3A). Non-toxigenic O1 strains, represented here by strains 12129 (1) and TM11079-80, are thought to have arisen from independent acquisitions of the O1 antigen region^12^. The O antigen region of 2012Env-9 and Env-390 formed a phylogenetic clade distinct from the PG clades and other non-toxigenic environmental isolates (Fig. 3B). When the divergent Amazonia strain (Brazil, 1987) is excluded, the Haitian strains cluster with the non-toxigenic O1 isolates with the date of divergence of all three O1 clades estimated at 1702 AD (SFig. 6). When taken into to consideration with the core genome phylogeny, these isolates may represent a 4^th^ inception of the O1 antigen phenotype beyond the three previously proposed. Furthermore, unlike non-PG group environmental O1 strains 12129(1) and TM11079-80, Haitian environmental strains 2012Env-9 and 2012Env-390 contain VPI-1, which encodes genes responsible for the biosynthesis of TcpA. Phylogenetically, VPI-1 regions of all three environmental Haitian strains (including non-toxigenic strain 2012EL-1786) are basal to the PG clade (Fig. 3C). Additionally, the organization of VPI-1 in the Haitian environmental strains is similar to both classical and El Tor biotypes and is structurally intact (Fig. 3D). These findings suggest that the Haitian environmental 2012Env-9 and 2012Env-390 strains diverged from the ancestral strain that gave rise to the PG clade. This also establishes that VPI-1 was not acquired through a recent horizontal gene transfer event during the 2010 Haitian cholera epidemic.

Chun *et al.* hypothesized that the progenitor of the PG clade possessed a type 1 core OS and O1 antigen gene cluster^12^. Our data supports this hypothesis since both Haitian non-toxigenic environmental O1 isolates possess these characteristics, with the O1 region located on an internal branch between PG and non-PG clades. Also, while 2012Env-9 and 2012Env-390 lack CTXϕ and its satellite phages, the presence of VPI-1 islands along with defective *dif* site and XerCD highlights the possibility of acquisition, replication and stabilization of the CTXϕ^19^. It is, therefore, likely that the Haitian strains represent a previously unknown phyletic lineage emerging from the ancestor of the PG clades, which has since lost CTXϕ and its satellite phages. The loss of these phages is not unlikely, as preservation and metabolic maintenance of CTXϕ and satellite phages, which contribute to virulence in humans, are costly during the aquatic phase of the microorganism’s life cycle^9^.

### Bayesian Coalescent Analysis

The origins of pandemic *V. cholerae* remain unclear due to difficulties in inferring deep-rooted phylogenies from genomes that may have undergone frequent lateral gene transfer, recombination, and site saturation^13^,^14^. Previous studies endeavoring to date the origins of pandemic cholera were also limited by the difficulty in estimating evolutionary rates from few available historical strains^13^,^20^. Until recently, the history of cholera prior to the divergence of the 6^th^ and 7^th^ pandemic clones in 1880 has been speculative^14^. However, the discovery and analysis of a strain from the 1849 Philadelphia cholera epidemic confirmed that the 2^nd^ and likely the 1^st^ pandemic were caused by a reemerging clone with a classical strain backbone^20^. This recent finding extended the demographic history of cholera to the first documented pandemic in 1817. Additionally, the analysis by Devault *et al.* estimated the ancestor of the PG clade to be 430 to 440 years ago; although, they suggest this is an underestimation due to site saturation and recombination^20^. Here, we provide evidence of an O1 lineage predating the PG clades and estimate a date ~500 years ago.

**Figure 4:**
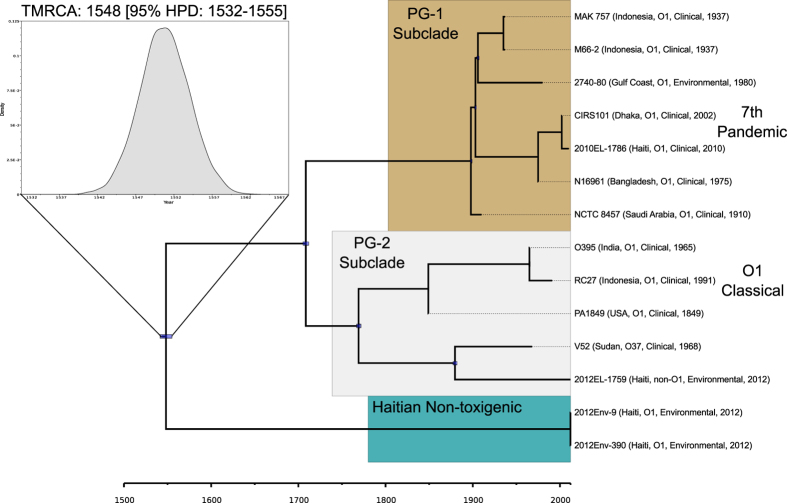
Bayesian maximum clade credibility tree (MCC) and dating of the most recent common ancestor (TMRCA) of toxigenic and non-toxigenic *V. cholerae* strains. Clades are labeled and annotated as in Fig. 2. The phylogeny was inferred from a ProgressiveMauve alignment of 14 strains that included 59,683 SNPs at sites conserved across all taxa. BEAST 1.8.2 was used to estimate TMRCA enforcing a strict molecular clock of 6.23 × 10^−4^ SNPs/SNP site/year and Bayesian Skyline demographic model. The marginal probability distribution for the root height is expanded from the highest posterior density (HPD) for the TMRCA of the Haitian non-toxigenic strains and the PG-1 and PG-2 subclades. Posterior probabilities are all above 80%. Blue node bars represent 95% HPD.

Bayesian molecular clock analysis was used to estimate the most recent common ancestor (TMRCA) of the Haitian environmental strains and the PG clades. Three isolates (Amazonia, 12129(1), and LMA3984-4) that were basal to the Haitian clade were excluded due to their level of divergence and the core genome analysis was repeated. The final core genome alignment of the remaining 14 strains included 59,683 SNPs at both inter- and intragenic sites conserved across all taxa. In this alignment, 2012Env-9 and 2012Env-390 only differed by two SNPs. The ML analysis was repeated and Path-O-Gen determined only moderate root-to-tip versus collection date correlation (0.56) (SFig. 7); therefore, similar to the approach by Devault and colleagues a strict molecular clock with a set rate of 6.23 × 10^−4^ SNPs/SNP site/year (adapted from^20^ – see methods) and Bayesian Skyline demographic model were enforced to date TMRCA. Despite the margin of error, the TMRCA was estimated at 1548 C.E. [95% HPD: 1532-1555] (Fig. 4). This finding suggests that this divergence predates the known history of cholera in Hispaniola and points to an earlier introduction.

### Environmental Persistence

To investigate how these nontoxigenic O1 strains may have survived for centuries in aquatic reservoirs in Haiti, we tested their ability to produce exopolysaccharide, which confers resistance to environmental stressors, as well as resistance to Vibriophage-mediated infection. Both 2012Env-9 and 2012Env-390 were able to convert to a rugose phenotype under rugose-permissive growth conditions, suggesting that these strains retained rugose structural and regulatory genes^5^. For reasons unknown, our repeated attempts to inactivate the *vpsA* gene in a rugose background failed; however, we were able to create an in-frame mutation in the *vpsA* gene in the smooth-phenotype background of both non-toxigenic strains. Data presented in Supplemental Figure 9 shows that both smooth and rugose variants of 2012Env-9 produced copious amounts of exopolysaccharide, promoting biofilm formation, which could in turn allow the strains to potentially evade environmental stressors and thereby enhance long-term persistence in the aquatic environment. As expected, the *vpsA* mutant was unable to shift to a rugose phenotype and produce biofilm. Both isolates were resistant to infection with any of seven environmental Vibriophages isolated from aquatic reservoirs in Haiti, all of which caused lysis in toxigenic O1 isolates of Haitian strains associated with the 2010 epidemic (Ali, unpublished data). Furthermore, *vpsA* mutants of 2012Env-9 and 2012Env-390 were also resistant to environmental Vibriophages infection, indicating that the exopolysaccharide produced by 2012Env-9 and 2012Env-390 rugose variants was not responsible for resistance to phage lysis. Taken together, our results suggest that 2012Env-9 and 2012Env-390 are well adapted for persisting in aquatic reservoirs by evading multiple environmental stressors and potential predators.

## Conclusions

The current work identifies a *V. cholerae* O1 strain possessing VPI-1, a type 1 OS, and orphaned remnants of the CTX element. These strains also lack the SXT/R391-family ICE found in 7^th^ pandemic strains including the Haitian epidemic clone represented by 2010EL-1786. Taken together, it is therefore likely that the non-toxigenic O1 Hatian environmental strains characterized here represent a previously unknown phyletic lineage emerging from the pre-pandemic ancestor of the PG clades, which has since lost CTXϕ and its satellite phages. *V. cholerae* is ubiquitous in the natural aquatic environment, where it can exist as free-living cells or attached to copepods or zooplankton^21^. Within this environment, extensive lateral gene transfer may occur resulting in a myriad of genotypic and phenotypic combinations. This results in the tremendous diversity observed among non-O1/O139 strains, as has been observed in Haiti by our group and others^4^,^6^. It was previously thought that non-toxigenic O1 strains (e.g., 12129 (1) and TM11079-80) arose from independent acquisitions of the O1 antigen region^12^. However, based on whole-genome assemblies of two non-toxigenic O1 strains isolated from the Haitian aquatic environment, we present evidence here of a pre-pandemic strain that clusters in the evolutionary tree within strains responsible for clinical cholera, supporting the hypothesis that the history of cholera in Hispaniola includes toxigenic strains that predate written records. Furthermore, the temporal and spatial variation in collection is consistent with a more widespread distribution of this strain in Haitian waterways. Non-O1/O139 isolates harboring the *tcpA* gene have frequently been recovered in Haitian aquatic environments^4^. As noted by Kahler and others, this feature is characteristic of environmental non-O1/O139 isolates found in cholera endemic countries^4^,^22^,^23^.

While an exact date of emergence of pandemic cholera remains elusive as a result of the combined effects of recombination, drift, selection, and the considerable time since divergence, estimates based on extant genomes provide relevant information regarding the demographic history of *V. cholerae.* In particular, our study benefits from the recent addition of the classical 1849 pandemic strain, as well as a formal assessment of substitution saturation and phylogenetic signal. Comparatively, the correlation coefficient of root-to-tip distance versus collection date was significantly better than previous analyses. Despite this, we found it appropriate to enforce a strong prior on the evolutionary rate, based on previous estimates, to improve the calibration of the molecular clock and TMRCA estimates.

The characterization of non-toxigenic O1 strains is essential to characterize drivers for the emergence of new hybrid toxigenic strains, a central concern for public health policies, as well as for our basic understanding of cholera evolution. The environmental strains described here represent suitable candidates for acquisition of virulence genes from the Haitian epidemic clone as they already possess VPI-1 that may act as a receptor for CTXϕ^24^. Additionally, the resistance for phage predation and the high-level production of surface polysaccharide suggests that these strains are well adapted for life in the Haitian aquatic environment and heightens concerns that if they acquire the cholera toxin producing CTXϕ, they could potentially establish themselves as a toxigenic strain persisting in the aquatic environmental reservoir.

## Material and Methods

### Isolation and characterization of *V. cholerae* strains

Non-toxigenic (i.e., ctx-negative) *V. cholerae* O1 strains, including 2012Env-9 and 2012Env-390, were isolated from water samples collected in La Salle (April, 2012) and Geffrarty (September, 2012), respectively, along the rivers in the Gressier region of Haiti. Water sampling locations, collection and processing of samples for *V. cholerae* O1 strains, serotyping, and PCR analysis for *V. cholerae* species specific and virulence specific genes have been described elsewhere^5^,^6^. In our previous analyses, 2012Env-9 and 2012Env-390 were negative for *ctxA/B* and *rstR*, but positive for *ompW*, *toxR*, and *tcpA*^CL^, suggesting the presence, at least in part, of VPI-1^6^.

### Biotyping, biofilm assay and phage susceptibility testing

Classical and El Tor biotypes of *V. cholerae* O1 can be differentiated using a variety of tests. We biotyped 2012Env-9 and 2012Env-390 O1 strains based on their susceptibility to polymyxin B (50U/ml), agglutination of chicken red blood cells, and reaction to Voges-Prausker (VP) test as described previously^25^. *V. cholerae* strains N16961 El Tor and O395 classical biotype were used as controls. A quantitative biofilm assay was performed on wild-type 2012Env-9 smooth, 2012Env-9*ΔvpsA* mutant, reverted 2012Env-9*ΔvpsA* mutant (*ΔvpsA*/pAA99), and rugose phenotype of 2012Env-9 as described previously^5^. As controls, we used N16961 smooth and rugose phenotypes. The conversion of smooth2012Env-9 to rugose2012Env-9 phenotype, creation of in-frame *vpsA* mutation in 2012Env-9 smooth background, and complementation of *vpsA* mutant was performed^5^. Vibriophages infection assay (plaque assay) was performed as described previously to determine whether Vibriophages can infect 2012Env-9 and 2012Env-390, and a 2012Env-9*ΔvpsA* mutant^26^.

### PacBio Sequencing and assembly

After gDNA isolation, purified genomic DNA from 2012Env-9 and 2012Env-390 was randomly fragmented using G-tubes (Covaris) to a nominal size of 10–12 kb and used for SMRTbell ligation. SMRT sequencing was carried out on a PacBio RS II using P5-C3 chemistry, magbead loading, and stage start instrument operation protocols. The initial 2012Env-9 assembly used the Hierarchical Genome Assembly Process (HGAP) developed by Chin *et al.*^27^. After base-call quality and read-length filtering, two SMRT cells provided approximately 800 Mb DNA sequence having N50 read length of 17.4 kb. The initial assembly produced three major contigs (2.7 Mb, 1.1 Mb, and 0.2 Mb) having 66x to 83x average DNA sequence coverage depth. Both ends of the 0.2 Mb contig overlapped the ends of the 2.7 Mb contig and were subsequently joined to form a final 2.9 Mb contig representing the larger 2012Env-9 chromosome. The individual chromosomes were circularized and the junctions polished for consensus sequence accuracy using the Quiver algorithm^27^. For 2012Env-390, two SMRT cells provided approximately 497 Mb of total sequence after filtering for read length and minimum read quality. The N50 read length of the assembly input sequence was 10.3 kb and the initial assembly produced three major contigs (2.85 Mb, 1.13 Mb, and 0.83 Mb) having 96x to 107x sequence coverage. The ends of 0.83 Mb contig overlapped with the ends of the 2.85 Mb contig and were joined to create a single 2.93 Mb contig representing the larger chromosome. The individual chromosomes were circularized and the consensus sequence polished for accuracy using the Quiver algorithm.

### Genomic comparison

Genomes of 2012Env-9 and 2012Env-390 were annotated with RAST online web server and Prokka v1.11 and annotations were assessed and merged^28^,^29^. Since similarity between both genomes was high (99% BLAST identity), 2012Env-9 was selected as the representative strain for genomic comparison (SFig. 8). Initially, core and accessory genomes were compared between 2012Env-9, O1 biotype El Tor strain N16961, and O1 classical biotype strain O395 using GET_HOMOLOGUES^30^. Genomic regions of interest (Supplementary Table 1) were compared between 2012Env-9 and O395. This analysis confirmed the presence of VPI-1 and the absence of the CTXϕ. Core and accessory genomes were then compared between representative published O1 and Non-O1/O139 genomes, specifically focusing on members of the phylocore genome subclades PG-1 and PG-2^12^ as well as other clinical and environmental non-toxigenic O1 strains. The recently published second pandemic classical biotype strain PA1849 as well as a non-O1/O139 Haitian environmental strain possessing VPI-1 and VPI-2 (2012EL-1759) were included in the analysis^20^,^31^. A single O37 isolate, V52, was also included due to its relationship to other members of the PG-2 clade, which include classical 6^th^ pandemic strains. Additionally, we assessed the presence of the SXT/R391-family antibiotic resistance element, a ~100-kb integrative conjugative element (ICE) that is predicted to have been acquired by pandemic cholera between 1978–1984^13^,^17^. A complete list of strains included in this study is provided in Supplementary Table 2. Since extensive lateral gene transfer has shaped the history of *V. cholerae*, we assessed genealogies of three distinct genomic regions. A gene alignment of the O antigen region (VC0241-VC0254, VC0259-VC0263 using the N16961 position), the oligosaccharide (OS) core region (VC0227, VC0234, VC0236, VC0239), and VPI-1 (VC0823-VC0834,VC0836-VC0845) were constructed from homologues regions identified by GET_HOMOLGUES and aligned with mafft^30^. The OS and O antigen region together constitute the major components of the lipopolysaccharide. These alignments were further assessed through phylogenetic analysis.

### Phylogenetic Analysis

Substitution saturation decreases phylogenetic signal and can significantly affect phylogenetic and coalescent analyses of deep-rooted phylogenies. To assess the potential impact of substitution saturation, we used three methods to assess substitution saturation in our data set. First, we visualized the distribution of single nucleotide polymorphism (SNP) densities across the core genome alignment and then plotted transitions and transversions versus Tamura and Nei 1993 (TN93) corrected genetic distance for 1 + 2 and 3 codon positions using DAMBE^32^. The Xia test, which is based on information-entropy, was then performed to statistically assess saturation^33^. Finally, TREE-PUZZLE was used to conduct likelihood mapping analysis on the core genome, OS region, VPI-1 region, and O1 antigen region alignments^34^.

To illustrate the relationship between O1 and non-O1/O139 *V. cholerae* strains, a phylogeny was inferred from homologous genes in the core genome using RAxML v.8.1.2 using the general time-reversible (GTR) nucleotide substitution model with gamma distributed rate variation among sites and 1000 bootstrap replicates^35^. This analysis was then repeated for clinical and environmental O1 strains. Three strains (12129(1), Amazonia, and LMA894-4) were found to be basal to the clade including 2012Env-9, 2012Env-390, and other O1 isolates. Therefore, to estimate the time of the most recent common ancestor (TMRCA) of 2012Env-9 and 2012Env-390, those three isolates were excluded and the core genome alignment of the 14 remaining isolates was assessed through Bayesian phylogenetic analysis. First, the correlation of root-to-tip distance versus year of isolation was assessed using Path-O-Gen^36^. The BEAST package v.1.8.2 was then used to reconstruct the demographic history and estimate the date of TMRCA. Constant, exponential, and Bayesian Skyline models enforcing strict and relaxed molecular clocks and GTR + G nucleotide substitution model were run for 500 million Markov-chain Monte-Carlo (MCMC) iterations with sampling every 50,000. Effective sampling size (ESS) values were assessed for each model to ensure proper mixing. Path sampling and stepping stone analysis were used to determine marginal likelihood estimates (MLE) for each model, which were then compared using Bayes factors. After careful comparison of the models and review of the correlation between root-to-tip distances and collection year (correlation coefficient = 0.56, R^2^ = 0.32), a strict molecular clock model enforcing a set evolutionary rate of 6.23 × 10^−4^ SNPs/SNP site/year was calibrated. This evolutionary rate was based on published values estimated from inter-pandemic cholera studies and is equivalent to the rate used by Devault *et al.* in their recent analysis of the PA1849 strain^13^,^20^.

## Additional Information

**How to cite this article**: Azarian, T. *et al.* Non-toxigenic environmental *Vibrio cholerae* O1 strain from Haiti provides evidence of pre-pandemic cholera in Hispaniola. *Sci. Rep.* **6**, 36115; doi: 10.1038/srep36115 (2016).

**Publisher’s note:** Springer Nature remains neutral with regard to jurisdictional claims in published maps and institutional affiliations.

## Supplementary Material

Supplementary Information

## References

[b1] Hendriksen R. S. *et al.* Population genetics of *Vibrio cholerae* from Nepal in 2010: evidence on the origin of the Haitian outbreak. MBio 2, e00157–11 (2011). 21862630 10.1128/mBio.00157-11 PMC3163938

[b2] Jenson D. & Szabo V. Cholera in Haiti and other Caribbean regions, 19th century. Emerg. Infect. Dis. 17, 2130–2135 (2011). 22099117 10.3201/eid1711.110958 PMC3310590

[b3] Azarian T. *et al.* Phylodynamic Analysis of Clinical and Environmental *Vibrio cholerae* Isolates from Haiti Reveals Diversification Driven by Positive Selection. MBio 5, e01824–14 (2014). 25538191 10.1128/mBio.01824-14 PMC4278535

[b4] Kahler A. M. *et al.* Surveillance for Toxigenic *Vibrio cholerae* in Surface Waters of Haiti. Am. J. Trop. Med. Hyg. ajtmh.13–0601, doi: 10.4269/ajtmh.13-0601 (2014). PMC4347365 25385860

[b5] Rahman M. *et al.* High-Frequency Rugose Exopolysaccharide Production by *Vibrio cholerae* Strains Isolated in Haiti. PLoS One 9, e112853 (2014). 25390633 10.1371/journal.pone.0112853 PMC4229229

[b6] Alam M. T. *et al.* Monitoring Water Sources for Environmental Reservoirs of Toxigenic *Vibrio cholerae* O1, Haiti. Emerg. Infect. Dis. Infect Dis 20, 356–363 (2014). 10.3201/eid2003.131293 PMC3944837 24571741

[b7] Hasan N. A. *et al.* Genomic diversity of 2010 Haitian cholera outbreak strains. Proc. Natl. Acad. Sci. USA 109, E2010–E2017 (2012). 22711841 10.1073/pnas.1207359109 PMC3406840

[b8] Jutla A. *et al.* Environmental factors influencing epidemic cholera. Am. J. Trop. Med. Hyg. 89, 597–607 (2013). 23897993 10.4269/ajtmh.12-0721 PMC3771306

[b9] Faruque S. M., Albert M. J. & Mekalanos J. J. Epidemiology, genetics, and ecology of toxigenic *Vibrio cholerae*. Microbiol. Mol. Biol. Rev. 62, 1301–1314 (1998). 9841673 10.1128/mmbr.62.4.1301-1314.1998 PMC98947

[b10] Karaolis D., Lan R. & Reeves P. The sixth and seventh cholera pandemics are due to independent clones separately derived from environmental, nontoxigenic, non-O1 *Vibrio cholerae*. J. Bacteriol. 177, 3191–3198 (1995). 7768818 10.1128/jb.177.11.3191-3198.1995 PMC177010

[b11] Meibom K. L., Blokesch M., Dolganov N. A., Wu C.-Y. & Schoolnik G. K. Chitin induces natural competence in *Vibrio cholerae*. Science 310, 1824–1827 (2005). 16357262 10.1126/science.1120096

[b12] Chun J. *et al.* Comparative genomics reveals mechanism for short-term and long-term clonal transitions in pandemic Vibrio cholerae. Proc. Natl. Acad. Sci. USA 106, 15442–15447 (2009). 19720995 10.1073/pnas.0907787106 PMC2741270

[b13] Mutreja A. *et al.* Evidence for several waves of global transmission in the seventh cholera pandemic. Nature 477, 462–465 (2011). 21866102 10.1038/nature10392 PMC3736323

[b14] Feng L. *et al.* A recalibrated molecular clock and independent origins for the cholera pandemic clones. PLoS One 3, e4053 (2008). 19115014 10.1371/journal.pone.0004053 PMC2605724

[b15] Hasan N. A. *et al.* Genomic diversity of 2010 Haitian cholera outbreak strains. Proc. Natl. Acad. Sci. USA 109, E2010–E2017 (2012). 22711841 10.1073/pnas.1207359109 PMC3406840

[b16] Baron S. *et al.* No Evidence of Significant Levels of Toxigenic *V. cholerae* O1 in the Haitian Aquatic Environment During the 2012 Rainy Season. PLoS Curr. 5 (2013). 10.1371/currents.outbreaks.7735b392bdcb749baf5812d2096d331e PMC3783635 24077904

[b17] Wozniak R. A. F. *et al.* Comparative ICE genomics: insights into the evolution of the SXT/R391 family of ICEs. PLoS Genet. 5, e1000786 (2009). 20041216 10.1371/journal.pgen.1000786 PMC2791158

[b18] Kamruzzaman M. *et al.* RS1 Satellite Phage Promotes Diversity of Toxigenic *Vibrio cholerae* by Driving CTX Prophage Loss and Elimination of Lysogenic Immunity. Infect. Immun. 82, 3636–3643 (2014). 24935981 10.1128/IAI.01699-14 PMC4187812

[b19] Faruque S. M. & Mekalanos J. J. Phage-bacterial interactions in the evolution of toxigenic *Vibrio cholerae*. Virulence 3, 556–565 (2012). 23076327 10.4161/viru.22351 PMC3545932

[b20] Devault A. M. *et al.* Second-Pandemic Strain of *Vibrio cholerae* from the Philadelphia Cholera Outbreak of 1849. N. Engl. J. Med., doi: 10.1056/NEJMoa1308663 (2014). 24401020

[b21] Huq A. *et al.* Ecological relationships between *Vibrio cholerae* and planktonic crustacean copepods. Appl. Environ. Microbiol. 45, 275–283 (1983). 6337551 10.1128/aem.45.1.275-283.1983 PMC242265

[b22] Chakraborty S. *et al.* Virulence genes in environmental strains of *Vibrio cholerae*. Appl. Environ. Microbiol. 66, 4022–4028 (2000). 10966424 10.1128/aem.66.9.4022-4028.2000 PMC92254

[b23] Faruque S. M. *et al.* Genetic diversity and virulence potential of environmental *Vibrio cholerae* population in a cholera-endemic area. Proc. Natl. Acad. Sci. USA 101, 2123–2128 (2004). 14766976 10.1073/pnas.0308485100 PMC357062

[b24] Waldor M. K. & Mekalanos J. J. Lysogenic conversion by a filamentous phage encoding cholera toxin. Science 272, 1910–1914 (1996). 8658163 10.1126/science.272.5270.1910

[b25] Kaper J., Morris, J. G., J. & Levine M. Cholera. Clin. Microbiol. Rev. 8, 48–86 (1995). 7704895 10.1128/cmr.8.1.48 PMC172849

[b26] Seed K. D. *et al.* Evidence of a dominant lineage of *Vibrio cholerae*-specific lytic bacteriophages shed by cholera patients over a 10-year period in Dhaka, Bangladesh. MBio 2, e00334–10 (2011). 21304168 10.1128/mBio.00334-10 PMC3037004

[b27] Chin C.-S. *et al.* Nonhybrid, finished microbial genome assemblies from long-read SMRT sequencing data. Nat. Methods 10, 563–569 (2013). 23644548 10.1038/nmeth.2474

[b28] Overbeek R. *et al.* The SEED and the Rapid Annotation of microbial genomes using Subsystems Technology (RAST). Nucleic Acids Res. 42, D206–D214 (2014). 24293654 10.1093/nar/gkt1226 PMC3965101

[b29] Seemann T. Prokka: rapid prokaryotic genome annotation. Bioinformatics 30, 2068–2069 (2014). 24642063 10.1093/bioinformatics/btu153

[b30] Contreras-Moreira B. & Vinuesa P. GET_HOMOLOGUES, a versatile software package for scalable and robust microbial pangenome analysis. Appl. Environ. Microbiol. 79, 7696–7701 (2013). 24096415 10.1128/AEM.02411-13 PMC3837814

[b31] Katz L. S. *et al.* Draft Genome Sequence of Environmental Vibrio cholerae 2012EL-1759 with Similarities to the *V. cholerae* O1 Classical Biotype. Genome Announc. 2 (2014). 10.1128/genomeA.00617-14 PMC4110763 25013135

[b32] Xia X. DAMBE: Software Package for Data Analysis in Molecular Biology and Evolution. J. Hered. 92, 371–373 (2001). 11535656 10.1093/jhered/92.4.371

[b33] Xia X., Xie Z., Salemi M., Chen L. & Wang Y. An index of substitution saturation and its application. Mol. Phylogenet. Evol. 26, 1–7 (2003). 12470932 10.1016/s1055-7903(02)00326-3

[b34] Schmidt H. A., Strimmer K., Vingron M. & von Haeseler A. TREE-PUZZLE: maximum likelihood phylogenetic analysis using quartets and parallel computing. Bioinformatics 18, 502–504 (2002). 11934758 10.1093/bioinformatics/18.3.502

[b35] Stamatakis A. *et al.* RAxML-Light: a tool for computing terabyte phylogenies. Bioinformatics 28, 2064–2066 (2012). 22628519 10.1093/bioinformatics/bts309 PMC3400957

[b36] Drummond A. J. & Rambaut A. BEAST: Bayesian evolutionary analysis by sampling trees. BMC Evol. Biol. 7, 214 (2007). 17996036 10.1186/1471-2148-7-214 PMC2247476

