# Supplementary material for: Non-toxigenic environmental Vibrio cholerae O1 strain from Haiti provides evidence of pre-pandemic cholera in Hispaniola
Source: Sci Rep. 2016 Oct 27;6:36115. doi: 10.1038/srep36115 (PMC5081557; doi:10.1038/srep36115)
Supplement: Supplementary Information [file srep36115-s1.pdf]

Non-toxigenic environmental *Vibrio cholerae* O1 strain from Haiti provides evidence of pre-pandemic cholera in Hispaniola

Taj Azarian[1¥]; Afsar Ali[1,2¥]; Judith A. Johnson[1,3]; Mohammad Jubair[1,2]; Eleonora Cella[1,4,5]; Massimo Ciccozzi[5,6]; David J. Nolan[1,3]; William Farmerie[7]; Mohammad H. Rashid[1]; Shrestha Sinha-Ray[1]; Meer T. Alam[1,2]; J. Glenn Morris Jr. [1,8*]; Marco Salemi[1,3*]

1. Emerging Pathogens Institute, University of Florida, Gainesville, USA; 2. Department of Environmental and Global Health, College of Public Health and Health Profession, University of Florida, Gainesville, Florida, USA; 3. Department of Pathology, Immunology and Laboratory Medicine, University of Florida, Gainesville, USA; 4. Department of Infectious, Parasitic and Immunomediated Diseases, Istituto Superiore di Sanità, Rome, Italy; 5. Department of Public Health and Infectious Diseases, Sapienza University of Rome, Rome, Italy; 6. University Hospital Campus Bio-Medico, Italy; 7. Interdisciplinary Center for Biotechnology Research, University of Florida, Gainesville, Florida, USA; 8. Department of Medicine, College of Medicine, University of Florida, Gainesville, Florida, USA

Author Contact

Taj Azarian
PO Box 103633, Gainesville, FL 32610, USA.
Tel +1 352 494 2011
taj.azarian@epi.ufl.edu

Afsar Ali
Box 100009, Gainesville, FL 32610, USA
Tel +1 352 273 7949
aali@epi.ufl.edu

Judith A. Johnson
PO Box 100009, Gainesville, FL 32610, USA.
Tel +1 352 273 9428
jajohnson@pathology.ufl.edu

Mohammad Jubair
Box 100009, Gainesville, FL 32610, USA
Tel +1 352 273 7949
jubair@epi.ufl.edu

Eleonora Cella
PO Box 103633, Gainesville, FL 32610, USA.
Tel/Fax +1 352 273 9567/8284
eleonora.cella@ufl.edu

Massimo Ciccozzi
Viale Regina Elena, 299, 00161 Rome, Italy
Tel +39-06-49903187
massimo.ciccozzi@iss.it

David Nolan
PO Box 103633
Tel +1 352 273 9567
djnolan@ufl.edu

William Farmerie
PO Box 103622
Gainesville, FL 32610
wgf2@ufl.edu

Mohammad H. Rashid
PO Box 100009, Gainesville, FL 32610, USA
Tel +1 352 253 7949
mhrashid@epi.ufl.edu

Shrestha Sinha-Ray
Box 100009, Gainesville, FL 32610, USA
Tel +1 352 273 7949
Shrestha@ufl.edu

Meer T. Alam
PO Box 100009, Gainesville, FL 32610, USA
Tel +1 352 253 7949
mtalam@epi.ufl.edu

*J. Glenn Morris, Jr.
PO Box 100009, Gainesville, FL 32610, USA
Tel +1 352 273 7526
jgmorris@epi.ufl.edu

*# Marco Salemi
PO Box 103633, Gainesville, FL 32610, USA.
Tel/Fax +1 352 273 9567/8284
salemi@pathology.ufl.edu

¥ Both authors equally contributed to the manuscript and thus would be considered as first authors.

* Drs. Morris and Salemi serve as joint senior authors of the manuscript

# Corresponding Author: Taj Azarian, Ph.D. MPH Taj.Azarian@epi.ufl.edu

Supplementary Material

Supplementary Table 1. List of isolates used in the analysis

Strain	Location	Type	Source	Year	Accession
2012EL-1759	Haiti	non-O1	Environmental	2012	JNEW01000001.1
2012Env-09	Haiti	O1	Environmental	2012	CP012997, CP012998
2012Env-390	Haiti	O1	Environmental	2012	CP013013, CP013014
2010EL-1786	Haiti	O1	Clinical	2010	CP003069, CP003070
LMA3984-4	Brazil	O1	Environmental	2007	NC_017269, NC_017270
CIRS101	Dhaka	O1	Clinical	2002	NZ_ACVW00000000
RC27	Indonesia	O1	Clinical	1991	ADAI01000045.1
TM11079-80	Brazil	O1	Clinical	1991	NZ_ACHW01000034, NZ_ACHW01000035
Amazonia	Brazil	O1	Clinical	1987	NZ_AFSV01000062, NZ_AFSV01000063
2740-80	Gulf Coast	O1	Environmental	1980	NZ_AAUT00000000.1
12129(1)	Australia	O1	Environmental	1980	NZ_ACFQ01000011, NZ_ACFQ01000012
N16961	Bangladesh	O1	Clinical	1975	NC_002505,NC_002506
V52	Sudan	O37	Clinical	1968	AAKJ02000055, AAKJ02000001
O395	India	O1	Clinical	1965	NC_009456, NC_009457
MAK757	Indonesia	O1	Clinical	1937	NZ_AAUS00000000.2
M66-2	Indonesia	O1	Clinical	1937	NC_012578, NC_012580
NCTC8457	Saudi Arabia	O1	Clinical	1910	NZ_AAWD00000000.1
PA1849	USA	O1	Clinical	1849	SRP029921

Supplementary Table 2. Genomic regions of interest

Region	Chr	Position	Description
O1	II	2,771,546-2,795,569	O1-antigen region
GI-1	II	1,043,291-1,055,071	Motility and chemotaxis
GI-2	II	1,248,952-1,252,534	Oxidative stress response
GI-3	II	1,435,685-1,440,550	Membrane proteins
GI-4	II	1,514,201-1,523,686	Carbohydrates (PTS system)
GI-5	I	1,005,832-1,011,520	Site-specific DNA-methyltransferase
GI-6	I	919,877-925,639	Putattive prophage
GI-7	I	664,079-669,638	Sodium-solute symporter
GI-8	I	486,113-489,350	Transposable element
GI-9	I	417,216-423,212	Autolysin sensor kinase/ABC-type transport system
GI-10	I	388,323-393,223	Integrase/Non-hemolytic enterotoxin lytic component L1
VPI-1	II	355,973-404,865	Vibrio pathogenicity island-1 (VPI-1)
VPI-2	II	1,449,205-1,505,734	Vibrio pathogenicity island-2 (VPI-2)
GI-11	I	1,012,862-1,045,566	Kappa prophage
GI-14	I	814,223-832,392	Hypothetical proteins
GI-21	II	689,569-722,653	Mu-like prophage
		766,615-799,585	Mu-like prophage
GI-23	II	2,499,645-2,522,558	Putative prophage
GI-24	II	2,825,203-2,840,525	Putative prophage(CRISPR-associate proteins)
CTX	II	1,114,655-1,123,257	Cholera toxin prophage (CTX)
CTX	I	560,562-567,519	Cholera toxin prophage (CTX)
TLC	II	1,123,876-1,138,038	Cryptic plasmid linke to CTX prophage

Supplementary Table 3. Phenotypic analysis of *V. cholerae* biotypes. Testing included susceptibility to polymixin B, chicken red blood cell agglutination test, and Voges-proskauer test.

**Strain**	**Serotype/biotype**	**Susceptibility to polymixin B (50U/ml)[a]**	**Agglutination with chicken red blood cells**	**Voges-proskauer**
N16961	O1/ El Tor	R	+	+
O395	O1/classical	S	-	-
2012Env-09	Hybrid biotype	R	-	+
2012Env-390	Hybrid biotype	R	-	+

[a]R=resistant; S=sensitive

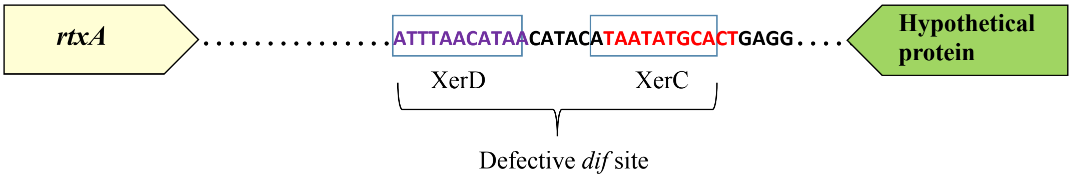

Supplementary Figure 1. Schematic representation of defective *dif* site in 2012Env-9 chromosome 1. Compared to N16961 El Tor strain, 2012Env-9 has a 21,151 bp absence of the CTXϕ and satellite phages, including RS1ϕ and TLCϕ. Instead, a 382 bp non-coding region is present between *rtxA* [VC1451 (N16961), NH62_20939 (2012Env-9)] and hypothetical protein [VC1479 (N116961), NH62_20940 (2012Env-9)]. This region has a 28 bp defective *dif* site that encompassing the binding sites for putative XerC and XerD recombinases.

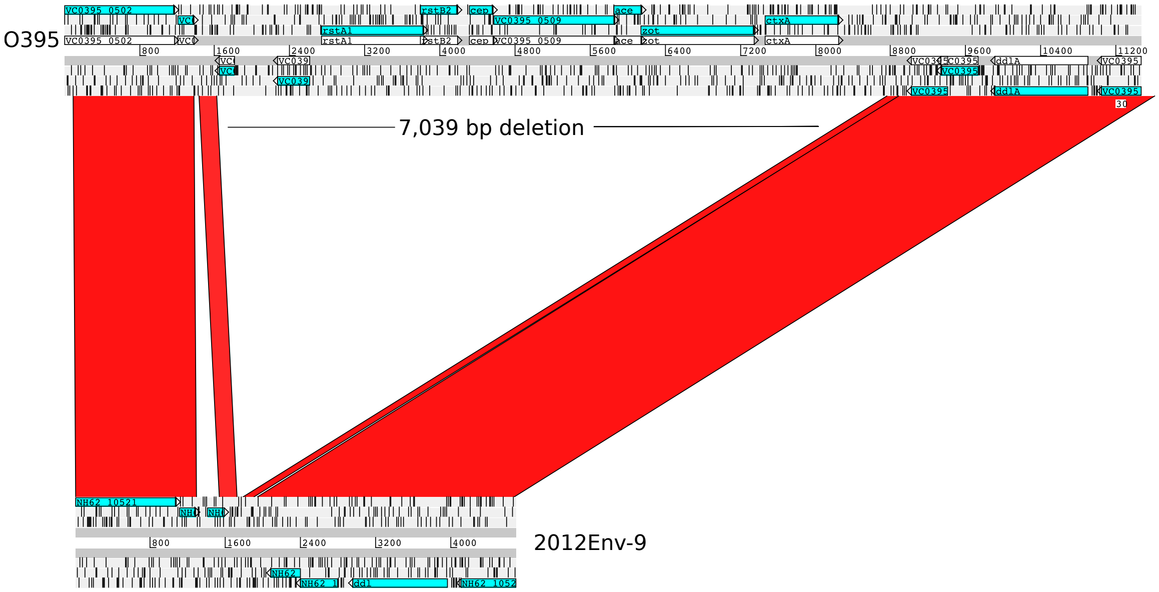

Supplementary Figure 2. BLAST sequence identity comparison of RS2 and CTXϕ regions of the O395 classical strain to homologous flanking regions of 2010Env-9. Regions of comparison include nucleotide positions 558,913-570,547 of chromosome 2 in the O395 genome (one of two locations of CTX in the classical strain) and nucleotide positions 601,084-605,767 of chromosome 1 in the 2012Env-9 genomes. Areas of red demonstrate high sequence identify (99-100%). The comparison, visualized in ACT v13.0.0, illustrates that while 2012Env-9 share homology in flanking regions, there is a 7,039 bp region containing RS2 and CTXϕ is absent in 2012Env-9. The deletion in this region includes CTXϕ core genes *rstA\B\R*, *cep*, *ace*, and *zot*, as well as cholera enterotoxin, A subunit (*ctxA*). A 245 bp insertion was present in 2012Env-9 that has 100% nucleotide identity to a transposase present in the RS2/CTX region of toxigenic strain VC06-18 (GenBank accession: KP768424).

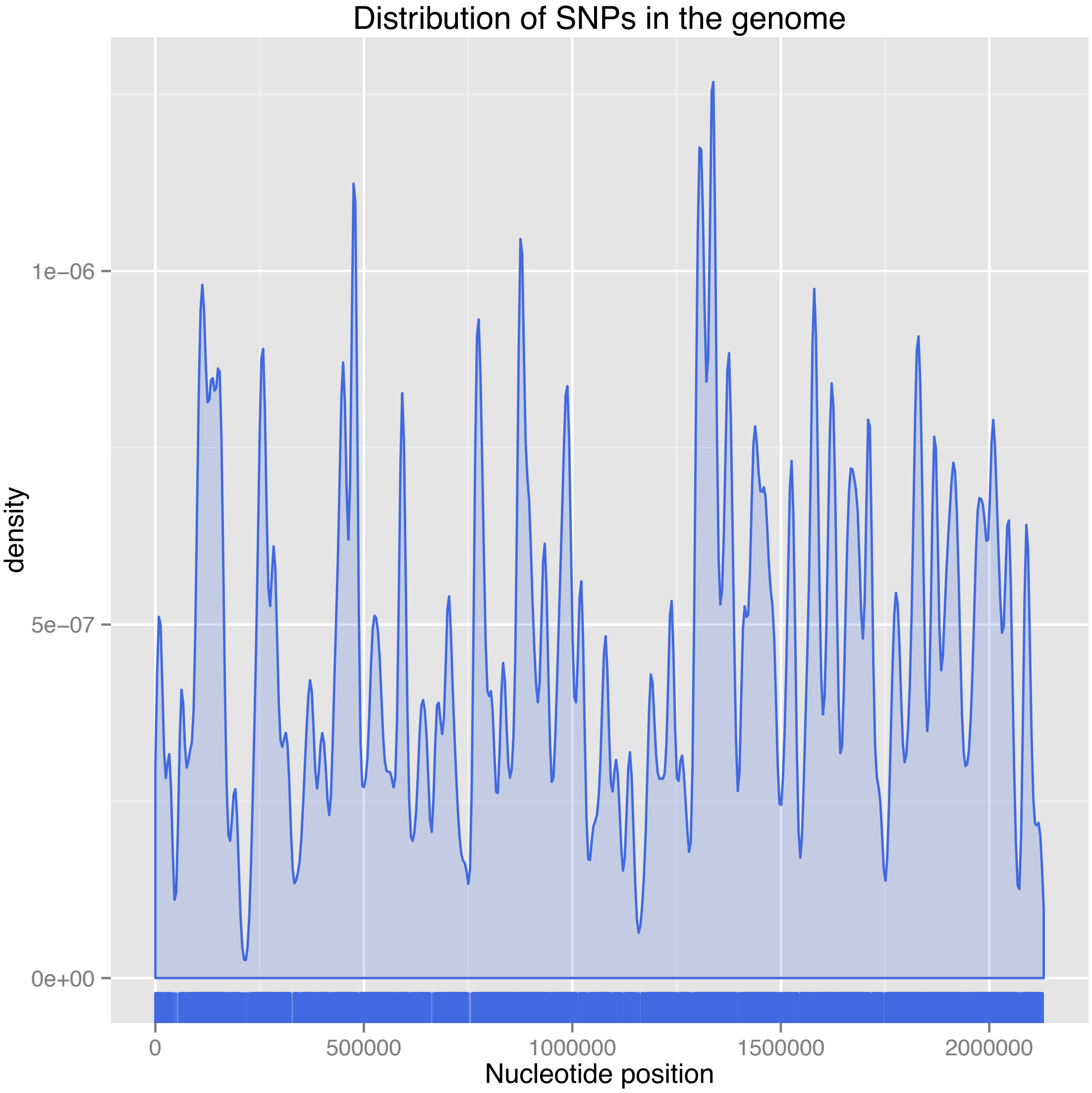

Supplementary Figure 3. Single nucleotide polymorphism density based on a core genome alignment of 14 *Vibrio cholerae* genomes including 2,163 genes with 2,130,441 bp.

A

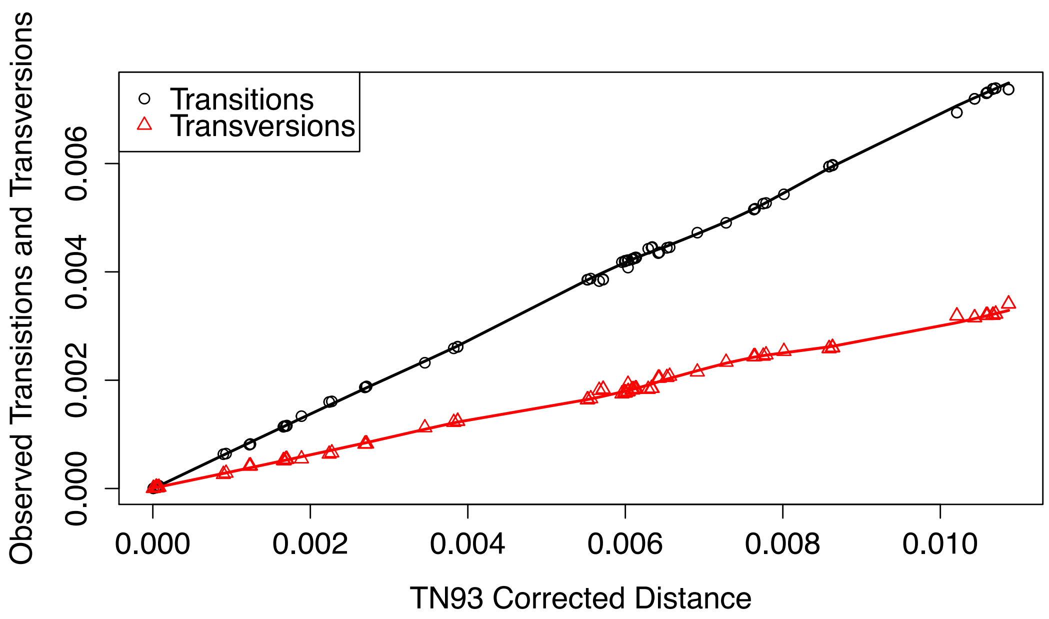

B

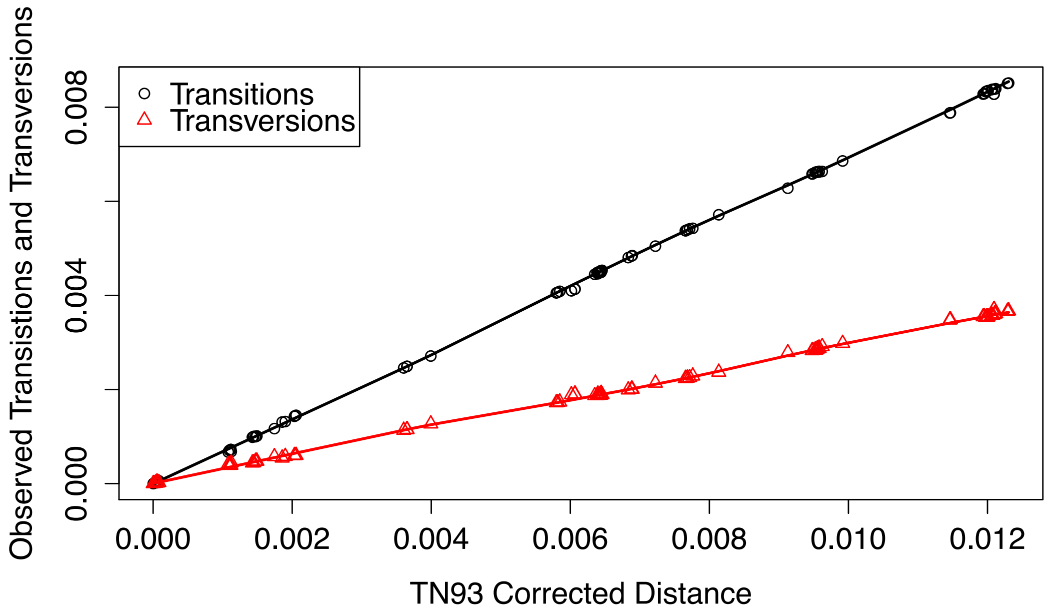

Supplementary Figure 4. Plotting the observed transition and transversions versus TN93 corrected genetic distance using DAMBE for the A) 1+2 codon position and B) 3 codon position. Consistent with absence of substitution saturation, transitions and transversions increase linearly with genetic distance, with transitions being greater than transversions.

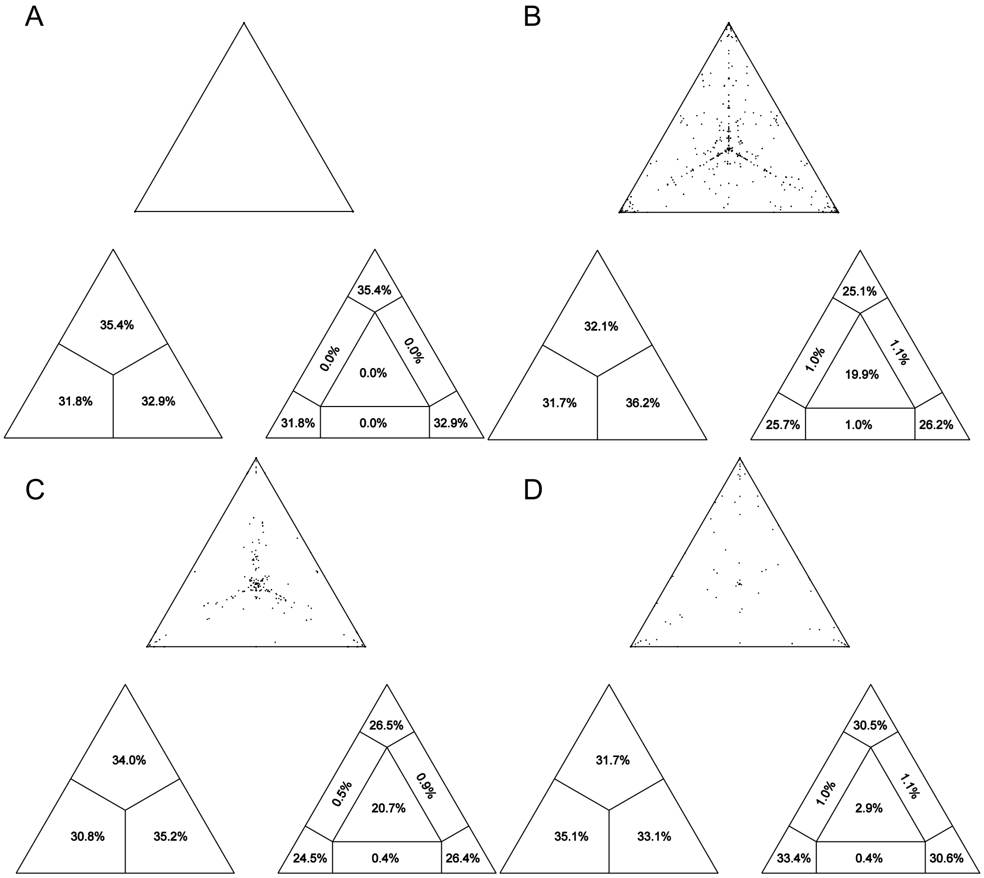

Supplementary Figure 5. Likelihood mapping analysis of core genome of A) 14 *V. cholerae* genomes, B) oligosaccharide (OS) region including four genes (4,383 bp) (VC0227, VC0234, VC0236, VC0239), C) O antigen region including 16 genes (24,517 bp) (VC0241-VC0254, VC0259-VC0263), and D) Vibrio Pathogenicity Island 1 (VPI-1) including 23 gene (19,503 bp) (VC0823-VC0834,VC0836-VC0845). Percentages of dots (topologies) in triangle corners represent fully resolved phylogenies, while the percentage in the center of the triangle represents a star-like genealogy indicating either the presence of recombination or conflicting phylogenetic signals. Greater than 50% of dots falling within the central area indicate substantial star-like signal.

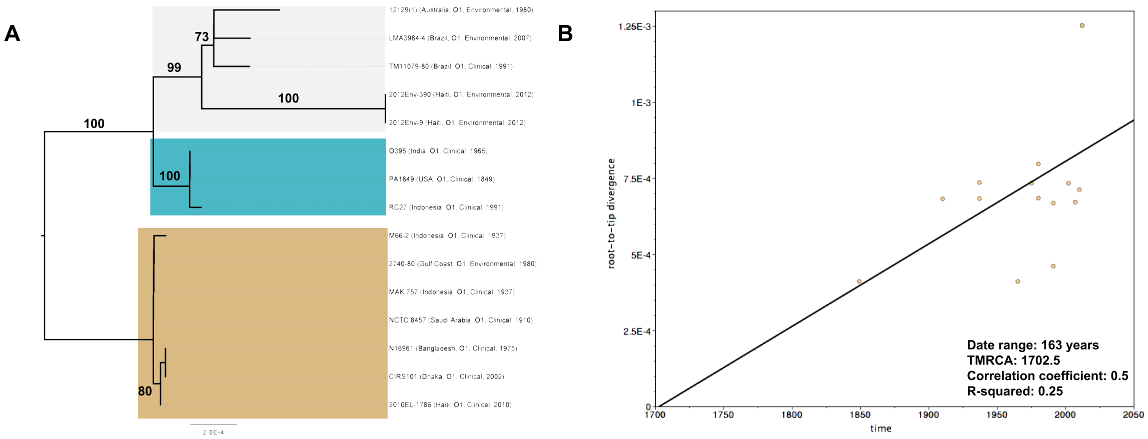

Supplementary Figure 6. Maximum likelihood analysis of O antigen region and Path-O-Gen analysis. A) The phylogeny was inferred from RAxML analysis of core genome alignment of 15 *V. cholerae* O1 strains including two Haitian non-toxigenic O1 environmental isolates. Bootstrap values are labeled on the branches and clades are colored based on the inferred independent acquisition of the O1 antigen region through horizontal gene transfer. B) Root-to-tip versus collection date analysis of *V. cholerae* O1 strains using Path-O-Gen. Path-O-Gen was also used to root the phylogeny in panel A and estimate the date of TMRCA or the O1 region.

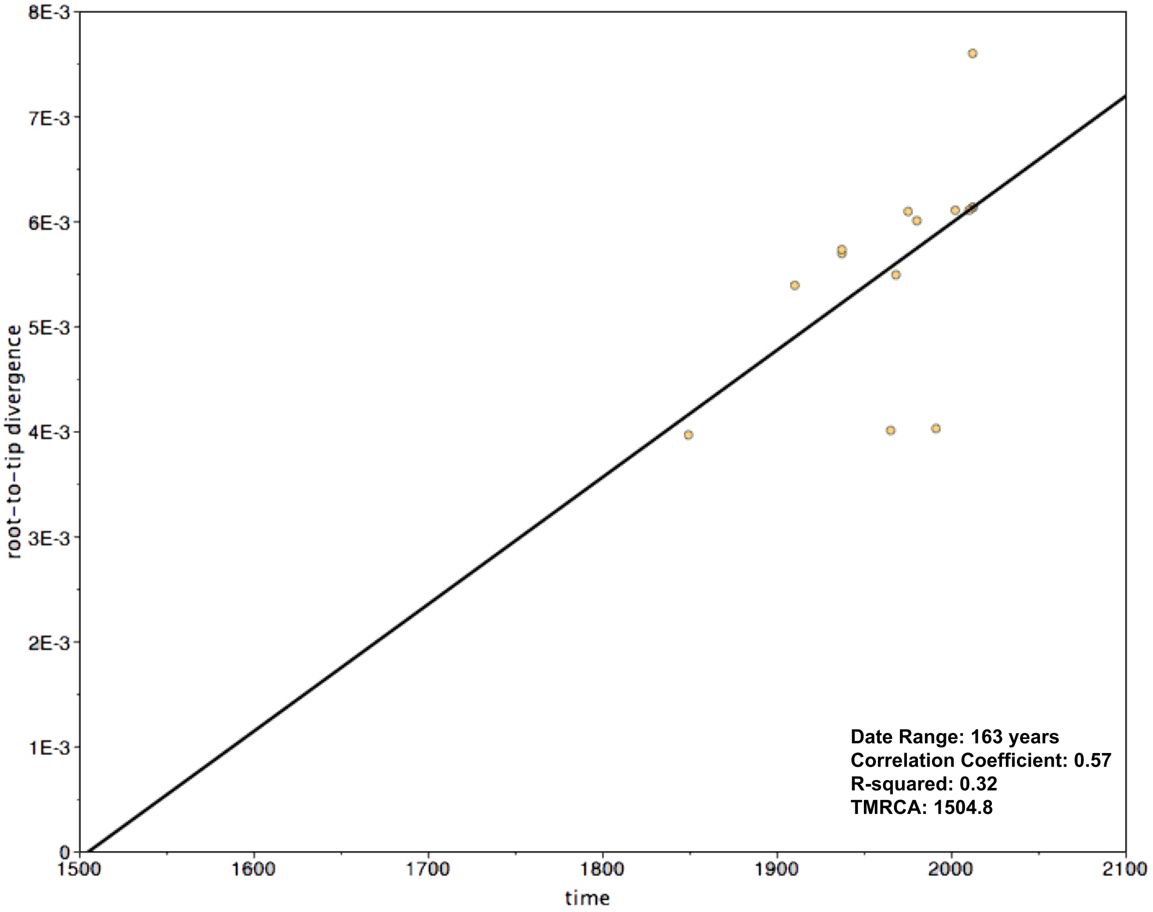

Supplementary Figure 7. Path-O-Gen analysis of root-to-tip distance versus year of collection for each isolate used in Bayesian phylogenetic analysis. This analysis was also used to root the maximum likelihood phylogeny.

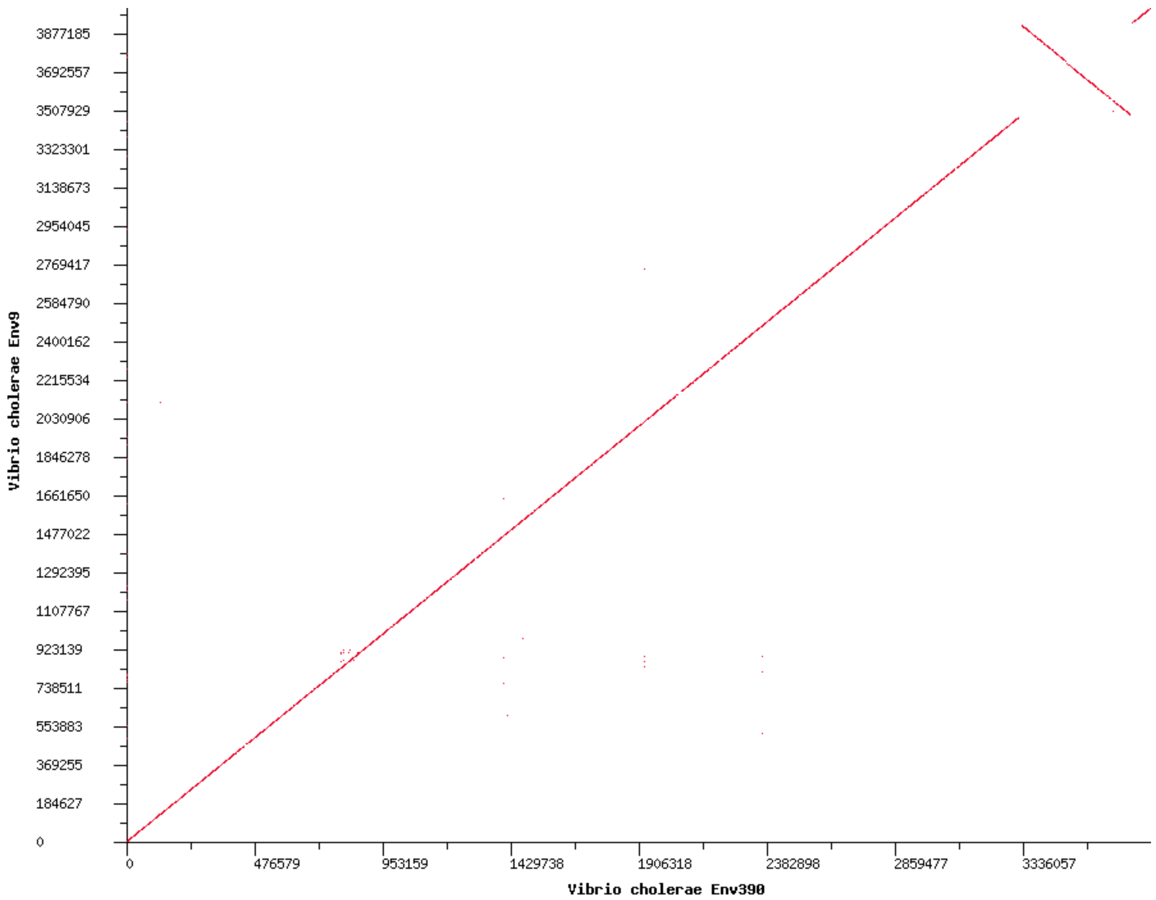

Supplementary Figure 8. Blast dot plot of both chromosomes of *Vibrio cholerae* 2012Env-9 vs. 2012Env-390. X- and y-axes represent the nucleotide in the concatenated chromosomes.

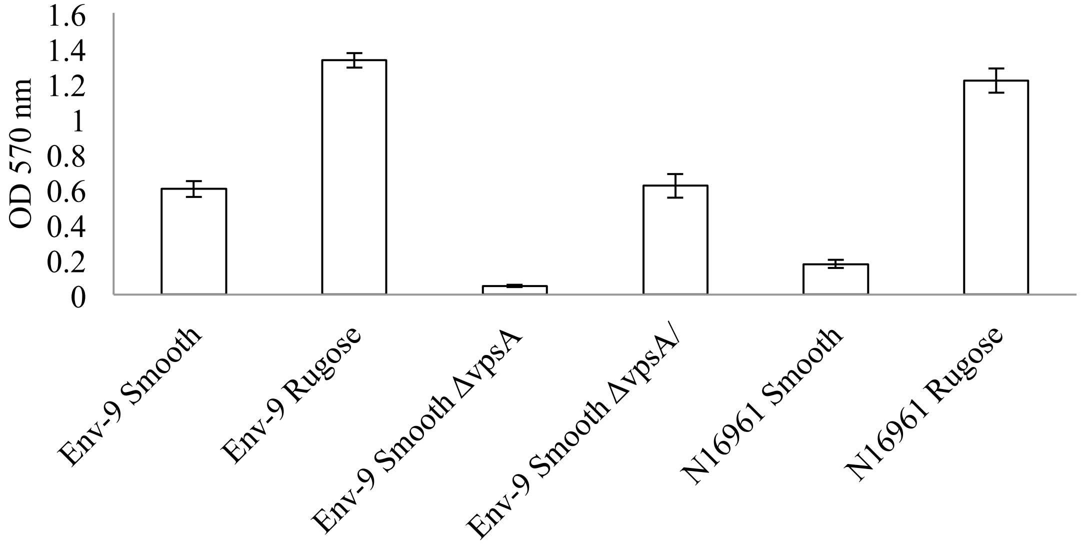

Supplementary Figure 9. Quantitative bioflim assay of non-toxigenic *Vibrio cholerae* O1 strains 2012Env-9 and 2012Env-390 with N16961. Assay compared both smooth and rugose producing phenotypes.
